# The utility of point-of-care ultrasound in critical care nephrology

**DOI:** 10.3389/fneph.2024.1402641

**Published:** 2024-10-03

**Authors:** Rogério da Hora Passos, Uri Adrian Prync Flato, Paula Rodrigues Sanches, Carolina Moraes Pellegrino, Ricardo Luiz Cordioli, Bruno Caldin Silva, Felipe Galdino Campos, Dalton de Souza Barros, Fernanda Oliveira Coelho, Bruno de Arruda Bravim, Thiago Domingos Corrêa

**Affiliations:** ^1^ Critical Care Department, Hospital Israelita Albert Einstein, São Paulo, Brazil; ^2^ Faculdade Israelita de Ciências da Saúde Albert Einstein, São Paulo, Brazil; ^3^ Critical Care Unit, Hospital CardioPulmonar, Salvador, Bahia, Brazil; ^4^ Critical Care Unit, Davita Tratamento Renal, Salvador, Bahia, Brazil

**Keywords:** point-of-care ultrasound, intensive care, acute kidney injury, nephrologist, VExUS, renal resistance index (RRI), AV fistula, nephrology

## Abstract

Point-of-care ultrasonography (POCUS) is gaining heightened significance in critical care settings as it allows for quick decision-making at the bedside. While computerized tomography is still considered the standard imaging modality for many diseases, the risks and delays associated with transferring a critically ill patient out of the intensive care unit (ICU) have prompted physicians to explore alternative tools. Ultrasound guidance has increased the safety of invasive procedures in the ICU, such as the placement of vascular catheters and drainage of collections. Ultrasonography is now seen as an extension of the clinical examination, providing quick answers for rapidly deteriorating patients in the ICU. The field of nephrology is increasingly acknowledging the value of diagnostic point-of-care ultrasound (POCUS). By employing multi-organ POCUS, nephrologists can address specific queries that arise during the diagnosis and treatment of patients with acute kidney injury. This approach aids in ruling out hydronephrosis and offers immediate information on hemodynamics, thereby consolidating patient data and facilitating the development of personalized treatment strategies.

## 1 Introduction

The incidence of acute kidney injury (AKI) requiring renal replacement therapy (RRT) has significantly increased over the years, affecting up to 15% of critically ill patients (1–3). Intradialytic hypotension (IDH) has been found to complicate 10%–70% of intermittent hemodialysis sessions, approximately 40%–60% of sustained, low-efficiency dialysis sessions, and 19%–43% of continuous renal replacement therapy treatments. While the association between intradialytic hypotension and adverse outcomes is unclear, studies on critically ill patients have shown a higher mortality rate and impaired renal recovery in those who experience IDH (4–6). Additionally, critically ill patients treated with vasopressors for shock often experience significant and prolonged relative hypotension, which is associated with poor kidney-related outcomes (7). There is no definitive evidence supporting the routine use of any specific intervention to prevent IDH, as its causes are multifactorial and include both the dialysis process itself and factors related to critically ill patients (8). The combination of decreased blood volume and impaired vascular resistance, along with reduced cardiovascular reserve, can lead to hemodynamic instability. Furthermore, the dialysis process has the potential to disrupt compensatory mechanisms, increasing the risk of hypotension. Point-of-care ultrasonography (POCUS) is now used in many clinical settings to enhance patients’ management. An assessment of predialytic cardiopulmonary profiles, defined based on sonographic findings, could facilitate IDH prediction and is an emerging part of critically ill patients’ bedside evaluation. Additionally, several other patient-related factors, such as baseline cardiac dysfunction, vascular tone, and impaired compensatory responses, can contribute to IDH. Therefore, bedside ultrasound for cardiovascular performance assessment serves as a valuable tool for conducting a comprehensive evaluation of patients requiring renal replacement therapy. In this review, we discuss the rationale behind nephrologists performing POCUS, explain the fundamental principles of focused ultrasonography, and provide our expert perspective on its effectiveness for delineating and comprehending the mechanisms of hypotension in patients undergoing RRT. We also discuss how POCUS can contribute to personalized resuscitation and improve patient outcomes.

## 2 The use of point-of-care ultrasound in the field of acute care nephrology

**Figure 1:**
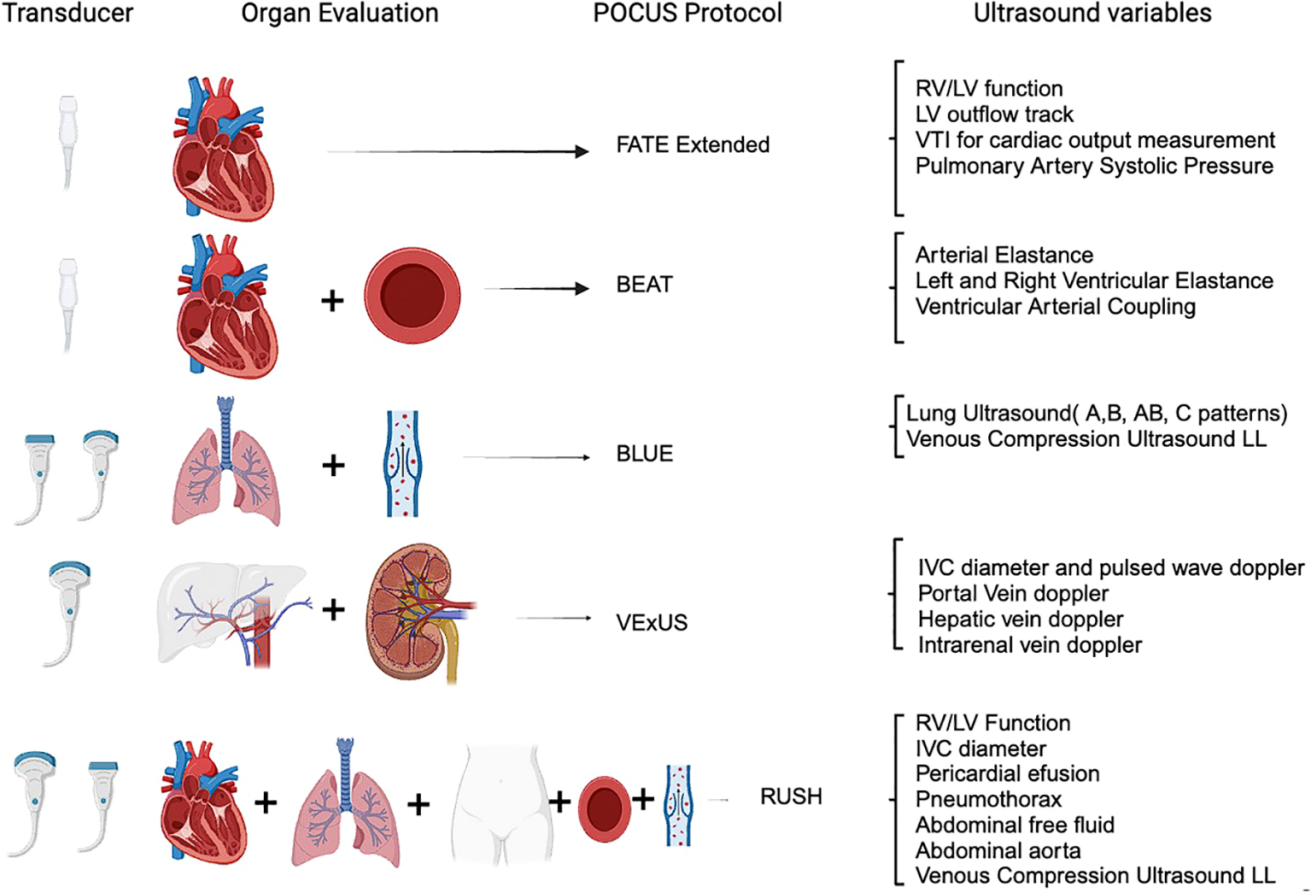
Point-of-care ultrasound protocols for acute care nephrology. RV, right ventricle; LV, left ventricle; VTI, velocity time integral; IVC, inferior vena cava; LL, lower limb; FATE extended (9), focus-assessed transthoracic echocardiography; BEAT (10), Browse the heart, Elastances, Assess volume status and Treat; BLUE (11), bedside lung ultrasound in emergency; RUSH (12), rapid ultrasound in shock examination; VExUS (13), venous excess ultrasound.

Caring for acutely ill patients with AKI who are undergoing RRT can be extremely difficult. The nephrologist is tasked not only with managing the kidney disease and dialysis procedure itself but also with addressing all the consequences that arise from the disrupted balance within the body. The use of POCUS has expanded significantly (**Figure 1**), and it has become an effective tool in diagnosing the cause of renal dysfunction, identifying pulmonary infiltrates, and assessing volume and acute circulatory failure in these patients (14, 15). Despite its clear benefits and acceptance in other medical fields, POCUS is still not widely used by nephrologists (16). The reasons for this are not clear, but it may be due to a lack of exposure and expertise acquired during training, time constraints for busy nephrologists, and a lack of standardized protocols for using POCUS. However, there has been a recent increase in interest among nephrologists, and POCUS training is now being incorporated into fellowship programs (17). The adoption of POCUS by nephrologists will provide them with a valuable tool that can greatly impact the management of AKI patients (18).

Point-of-care ultrasound (POCUS) is increasingly recognized as a vital tool in managing unstable medical conditions, offering rapid diagnostic insights that can significantly impact patient care outcomes. However, proficiency in performing ultrasound across various anatomical regions involves a notable learning curve that affects its effective utilization. Achieving competence in POCUS requires comprehensive training and continual practice to master image acquisition, interpretation, and integration into clinical decision-making (19, 20).

The learning curve for ultrasound proficiency varies depending on the body district being examined. For example, abdominal ultrasound necessitates expertise in visualizing organs through different acoustic windows while navigating challenges such as bowel gas interference (21). In contrast, mastering cardiac ultrasound requires precise probe positioning and advanced interpretation skills to assess cardiac chamber dimensions, valve function, and hemodynamics accurately (22).

Training programs tailored to POCUS encompass diverse educational approaches, including didactic sessions, hands-on workshops, simulation-based training, and supervised clinical practice. These programs are essential to ensure that clinicians acquire and maintain proficiency in ultrasound techniques across varied clinical scenarios (23). Competency assessments and quality improvement measures, such as peer review and proficiency evaluations, further enhance the reliability and clinical impact of POCUS in acute and critical care settings (24).

In conclusion, while POCUS enhances diagnostic capabilities in unstable conditions, addressing the learning curve across different body districts is essential for optimizing its clinical utility. Continuous training and proficiency assessments are crucial to ensure accurate and effective use of ultrasound, thereby improving patient outcomes in diverse clinical contexts.

## 3 Lung ultrasonography for nephrologists

Nephrologists and AKI patients struggle to control volume overload and its complications. Traditional signs of volume overload in physical examination, such as rales and edema, are not reliable indicators of pulmonary congestion (25). The reliable detection of pulmonary congestion holds the potential to predict the need for additional ultrafiltration (26). From a technical perspective, the appearance of a normal lung structure is hindered by reverberation artifacts caused by the difference in density between skin and soft tissue (water density) and the alveolar sac (air density) in ultrasound images (27). In healthy individuals, the lung exhibits a pattern of horizontal reflections called the A-line pattern. As pulmonary congestion increases, this pattern shifts to a vertically oriented B-line pattern. B-lines manifest as hyperechoic lines extending to the ultrasound field’s edge, moving synchronously with respiration. Quantifying pulmonary congestion involves counting B-lines across multiple intercostal spaces, with the eight-zone anterior lung ultrasound being the most validated method for research (28). The more B-lines are counted, the greater the pulmonary congestion, which correlates with extravascular lung water (29). Quantitative lung ultrasound has shown good reliability and agreement compared to ultrasound transducers. The optimal images are obtained by setting the focal depth at the pleural line, increasing gain in the far field, and turning off harmonics. Lung ultrasound surpasses physical examination and chest X-ray in predicting acute cardiogenic pulmonary edema (30). It is also sensitive in detecting pleural effusions, which appear as anechoic structures between the lung and diaphragm. Lung ultrasound can easily be taught using a remote web-based application.

**Figure 2:**
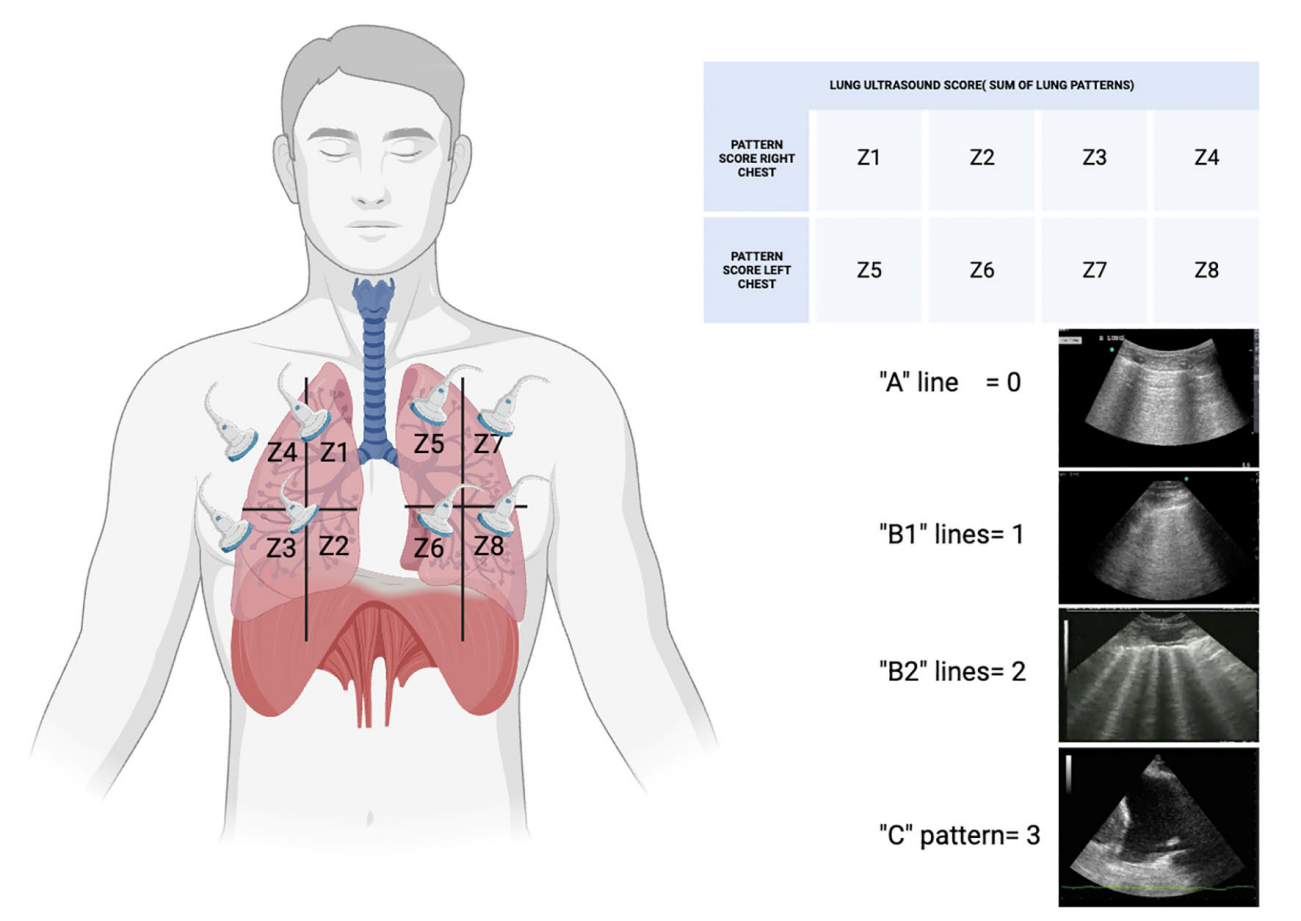
Diagram of the lung ultrasound score (LUS) adapted from Volpiceli et al. (28). Score grade from 0 to 24; the higher the score, the more severe the lung disease. LUS is a useful diagnostic tool in monitoring treatment and prognostic factors. Z, zone. The “B-lines” observed in lung ultrasonography are reverberation artifacts that indicate the presence of alveolar interstitial syndrome. The lines can be classified into various profiles according to the therapeutic context. For example, a “B1” profile could indicate a certain quantity and arrangement of B-lines that imply a specific pathological condition, while a “B2” profile could indicate a more severe or extensive interstitial disease. A “C” profile may indicate the existence of consolidation, indicating an alternative underlying condition such as pneumonia or atelectasis.

The lung ultrasound score (LUS) is a tool that has gained traction among nephrologists due to its utility in assessing and managing patients with kidney diseases. This scoring system is based on the quantification of B-lines using a quick eight-zone protocol (**Figure 2**) that can be completed in under 2 min (31). In practice, the lung is divided into eight zones, and each zone is scored based on the number and intensity of B-lines that are observed. The cumulative score provides a semiquantitative assessment of lung water, which correlates with the degree of pulmonary congestion. This method offers a more nuanced and precise approach to fluid management compared to traditional methods like physical examination or chest X-ray, particularly in dialysis patients, where an accurate assessment of dry weight is crucial. It is non-invasive and radiation-free and can be performed at the bedside with portable ultrasound machines. This makes it an ideal tool for real-time monitoring and decision-making in acute care settings. However, the accuracy of LUS depends on the operator’s skill and experience, highlighting the need for adequate training and standardization in its application.

When discussing lung ultrasound, it is crucial to elucidate the relationship between extravascular lung water (EVLW) and B-lines, particularly for optimizing dialysis sessions. Nephrologists have effectively utilized B-line assessments to enhance dialysis treatment, as evidenced by several key studies. For instance, Mallamaci et al. demonstrated the detection of pulmonary congestion via chest ultrasound in dialysis patients, highlighting the clinical utility of this method (32). Moreover, Noble et al. provided significant insights into the time course for the resolution of EVLW in patients undergoing hemodialysis, emphasizing the role of ultrasound in effectively monitoring and managing fluid status (33).

Lung ultrasound offers several advantages, particularly when compared to cardiac ultrasound. First, it is non-invasive, making it safer and more comfortable for patients. The method allows for frequent repetitions, enabling continuous monitoring of a patient’s condition without the risks associated with radiation exposure. Lung ultrasound also provides real-time results, facilitating immediate clinical decision-making, which is crucial in managing fluid status in dialysis patients (28). Furthermore, it is relatively low cost and can be performed at the bedside, making it accessible in various healthcare settings (29).

However, lung ultrasound does have limitations. The accuracy of the results is highly dependent on the operator’s skill and experience, necessitating thorough training and proficiency in interpreting ultrasound images (11). Variability between operators can lead to inconsistencies in diagnosis and assessment. Additionally, while lung ultrasound is excellent for detecting B-lines and assessing EVLW, it may be less effective in differentiating between various causes of pulmonary congestion, thus requiring supplementary diagnostic methods (34).

Despite these challenges, the application of chest ultrasound in dialysis patients offers substantial benefits. It provides a practical, effective, and accessible approach to the assessment and management of pulmonary congestion, ultimately improving patient outcomes and optimizing dialysis sessions. By integrating lung ultrasound into routine practice, clinicians can enhance their ability to monitor fluid status and tailor dialysis treatments more precisely, thereby enhancing overall patient care.

## 4 Cardiovascular ultrasonography for nephrologists

The main goal of basic cardiac ultrasonography is to provide succinct and targeted qualitative assessments that aid in guiding management decisions. This non-invasive imaging technique is particularly valuable in assessing left ventricular hypertrophy, cardiac chamber sizes, valvular heart disease, and systolic and diastolic function. It also plays a crucial role in evaluating fluid status and cardiac output, which are essential in managing fluid overload, a frequent challenge in dialysis patients. Moreover, regular cardiac ultrasonography can help in the early detection of cardiovascular diseases, allowing for timely intervention. The cognitive skills needed include assessing the overall size and function of the left ventricle, distinguishing between segmental and global wall motion abnormalities, evaluating the size and function of the right ventricle, and identifying severe valvular dysfunction using color Doppler (35). Basic cardiac ultrasonography helps in accurately categorizing shock and identifying the potential life-threatening causes of shock; shock is not uncommon in critically ill patients evaluated by nephrologists (36). Cardiac ultrasonography can quickly determine if there is evidence of hypovolemia, a pericardial effusion, or cardiac tamponade. The presence of an enlarged right ventricle may indicate acute cor pulmonale and impending right ventricular failure (37).

## 5 POCUS in hemodynamic instability during acute kidney injury and acute renal replacement therapy

Hemodynamic instability is a prevalent condition in critical illness and can have a significant impact on patient outcomes in the ICU. This instability can interfere with tissue perfusion and oxygen delivery, leading to multi-organ dysfunction. AKI is also frequently observed in critically ill patients and can further aggravate patient outcomes. Hypotension and AKI (38) are often associated, through direct and indirect mechanisms, with both conditions potentially stemming from a single underlying cause of organ damage. Approximately 10%–20% of ICU patients with AKI require acute RRT, and their expected mortality rate is nearly 50% (38). The epidemiological and pathophysiological correlation between hypotension and RRT is acknowledged although a clear definition of hemodynamic instability during RRT remains elusive (39). Reports on critically ill patients indicate that IDH is linked to increased mortality and compromised renal recovery. POCUS assessment can provide nephrologists with hemodynamic parameters that can be targeted and integrated into organ perfusion surrogates to potentially yield the best results (39–43). The utilization of POCUS in revealing these mechanisms holds significant implications for the management of patients, as reducing ultrafiltration may not always be the optimal course of action, particularly in instances of substantial fluid overload (44).

### 5.1 Preload dependence and POCUS

Ultrasound has emerged as a crucial tool for assessing volume status and response to volume resuscitation in critically ill patients. Its non-invasive nature and low cost make it an attractive option, and clinicians can perform repeat ultrasounds at the patient’s bedside as needed. Extensive research has focused on the elasticity of large veins, including the IVC and internal jugular vein, to determine standardized parameters for fluid responsiveness, such as the collapsibility index (45). These parameters are linked to traditional predictors of fluid responsiveness, such as central venous pressure (46). Recent studies have shown that large arteries, such as the common carotid artery, are also dynamically compliant and can predict fluid responsiveness (47). However, it is important to note that ultrasound interpretation is operator-dependent. To address this issue, standardized parameters like angulation probe and corrected flow time can be calculated using ultrasound, potentially minimizing discrepancies in image interpretation among providers (48).

### 5.2 Ventricular–arterial coupling and POCUS

**Figure 3:**
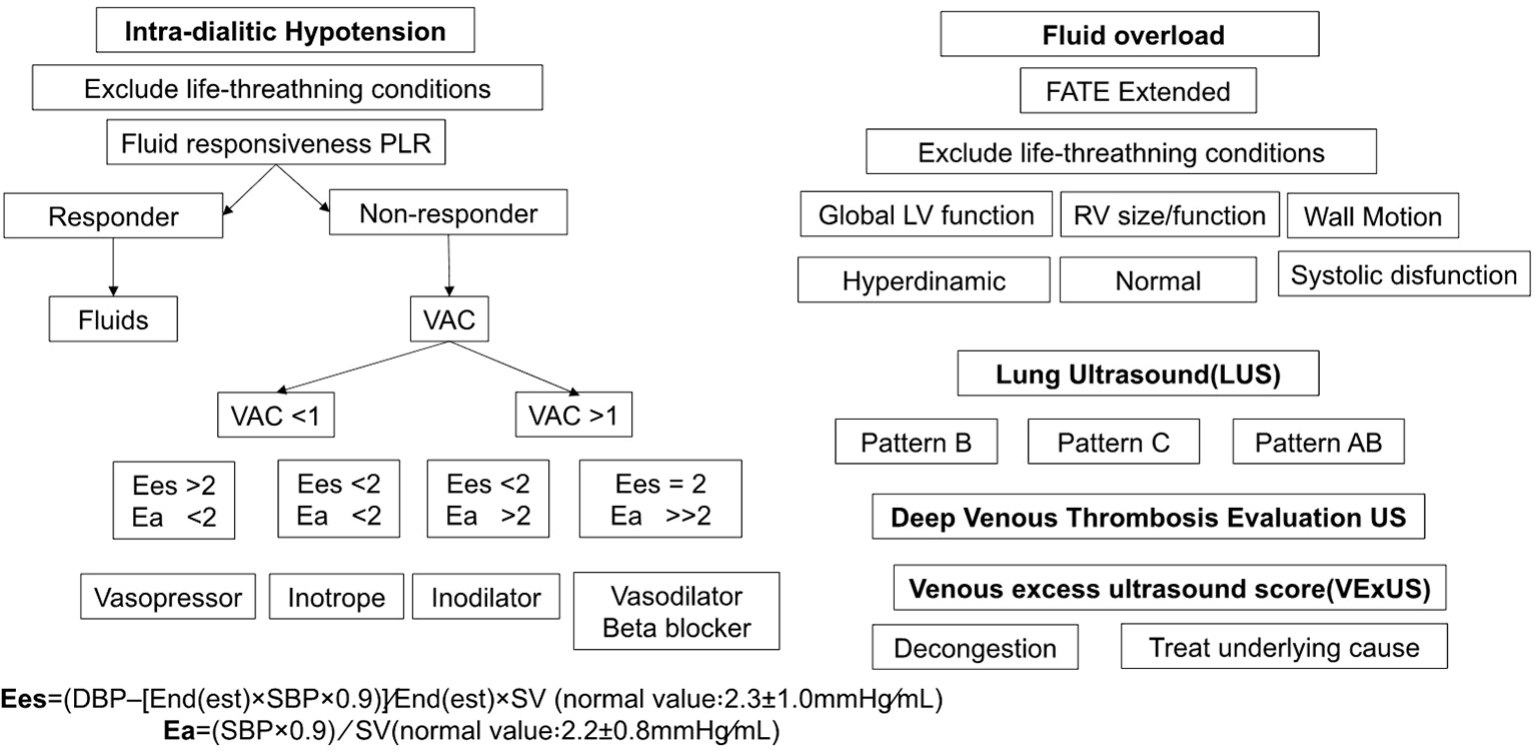
Resuscitation algorithm based on optimizing intradialytic hypotension and fluid overload in critically ill patients undergoing renal replacement therapy. PLR, passive leg raising; VAC, left ventricular–arterial coupling; *E*_a_, effective arterial elastance; *E*_es_, left ventricular effective end-systolic elastance; FATE, focus-assessed transthoracic echocardiography; LV, left ventricle; RV, right ventricle; US, ultrasound.

The idea that optimal cardiovascular performance is achieved when the heart and arterial system are coupled has been well-established through various studies (49). When the heart pumps blood into the vascular tree at a rate and volume that matches the arterial system’s ability to receive it, both cardiovascular performance and its associated cardiac energetics are optimized. Deviations from this optimal state, such as high or low contractility or arterial tone, can lead to cardiac failure, independent of other disease processes. Ventricular–arterial coupling (VAC) analysis quantifies the optimal matching of the left ventricular workload and the arterial system, with minimal changes in left ventricular (LV) pressure and the complete transfer of mechanical energy from the ventricle to the arterial system (50). The role of VAC in managing critically ill patients with severe hemodynamic instability and shock is increasingly being recognized. VAC is calculated as the ratio of arterial elastance (*E*_a_) to ventricular elastance (*E*_es_), proposed by Suga (51) as a measure of cardiovascular mechanical efficiency and the interaction between cardiac performance and vascular function. The *E*_a_/*E*_es_ ratio is a reliable and effective measure of cardiovascular performance, with optimal efficiency achieved when the ratio is near 1. VAC is an effective index of LV’s mechanical performance and dynamic modulation of the cardiovascular system (52) and reflects cardiac energetics. The balance between myocardial oxygen consumption and mechanical energy required for cardiac work is optimal when the heart and peripheral vascular system are coupled. The area of the LV pressure–volume (P–V) loop during a single cardiac cycle represents the total mechanical energy of the heart during that beat and correlates linearly with myocardial oxygen consumption. Understanding VAC requires knowledge of the determinants *E*_a_ and *E*_es_ and their bedside measurement in critically ill patients. LV contractile function can be evaluated using the relationship between end-systolic pressure (ESP) and end-systolic volume (**Figure 3**). Non-invasive measurement approaches for measuring VAC have been developed (53), with the modified single-beat method being validated against the invasive measurement of *E*_es_. This method utilizes echocardiographic measures of LV end-diastolic and end-systolic areas, LVEF, stroke volume, pre-ejection time, and systolic time interval, coupled with systolic and diastolic arterial pressure measurements. *E*_a_ is calculated as ESP/stroke volume or 0.9 × systolic arterial pressure/stroke volume. If the patient experiences hypotension during hemodialysis despite volumetric resuscitation, assessing parameters for VAC (vascular access compression) becomes crucial for guiding further interventions like inotropes or additional volume or vasopressors. These bedside measures are essential in evaluating VAC in critically ill patients. The resuscitation algorithm for patients on RRT is dynamic, mandating ongoing reassessment and adjustment based on the patient’s changing clinical condition. This highlights the need for a balanced approach to fluid management and hemodynamic support to enhance patient outcomes.

### 5.3 Myocardial function and POCUS

**Figure 4:**
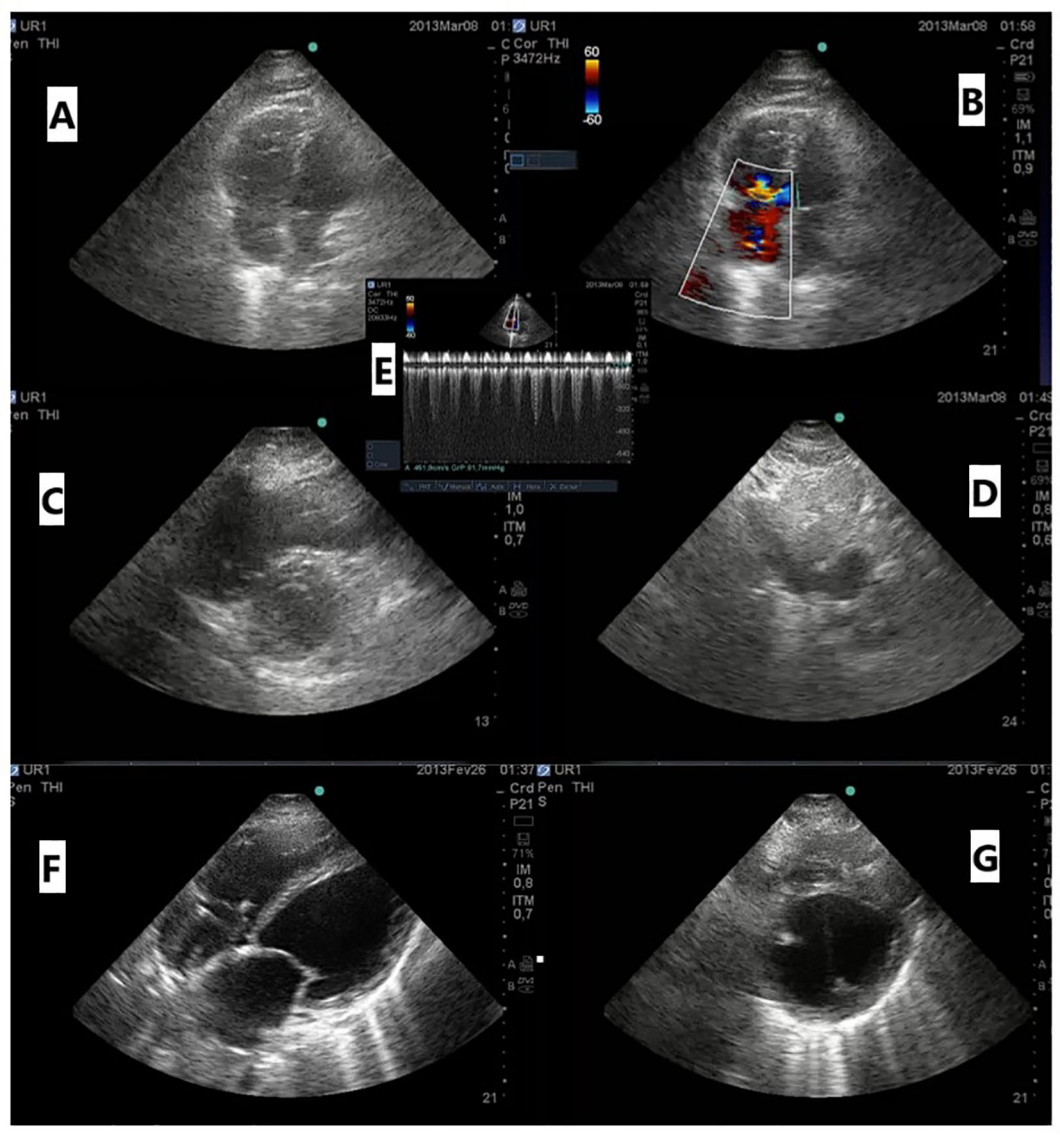
**(A)** Apical four-chamber (A4C) view with a dilated right ventricle (RV); **(B)** color Doppler of tricuspid regurgitation A4C; **(C)** parasternal short-axis view (PSA) showing an enlarged RV with a flattened interventricular septum; **(D)** dilated inferior vena cava; **(E)** continuous wave Doppler of tricuspid regurgitation showing estimated pulmonary systolic pressure of 96 mmHg in a patient with acute pulmonary embolism; **(F)** subcostal view with dilated left chambers; **(G)** PSA showing an enlarged left ventricle with severe systolic dysfunction related to myocarditis.

Several studies have been conducted to evaluate the accuracy of left ventricular assessment by non-cardiologists in detecting left ventricular systolic dysfunction after various training programs (54–56). These studies (57) have demonstrated good sensitivity in determining left ventricular function at the extreme ends, such as assessing a left ventricular ejection fraction (LVEF) above or below 50% (sensitivity 74%–95%) and an LVEF above or below 30% (sensitivity 100%). This indicates that even with limited training, physicians, residents, and medical students can accurately differentiate between normal and abnormal and severely and non-severely impaired left ventricular systolic function and determine the specific degree of left ventricular dysfunction (**Figure 4**). It has been demonstrated that focused cardiac ultrasound (58), even in the hands of novice users, is superior to clinical examination by experts in identifying cardiac abnormalities.

### 5.4 Systemic venous congestion and renal resistive index

**Figure 5:**
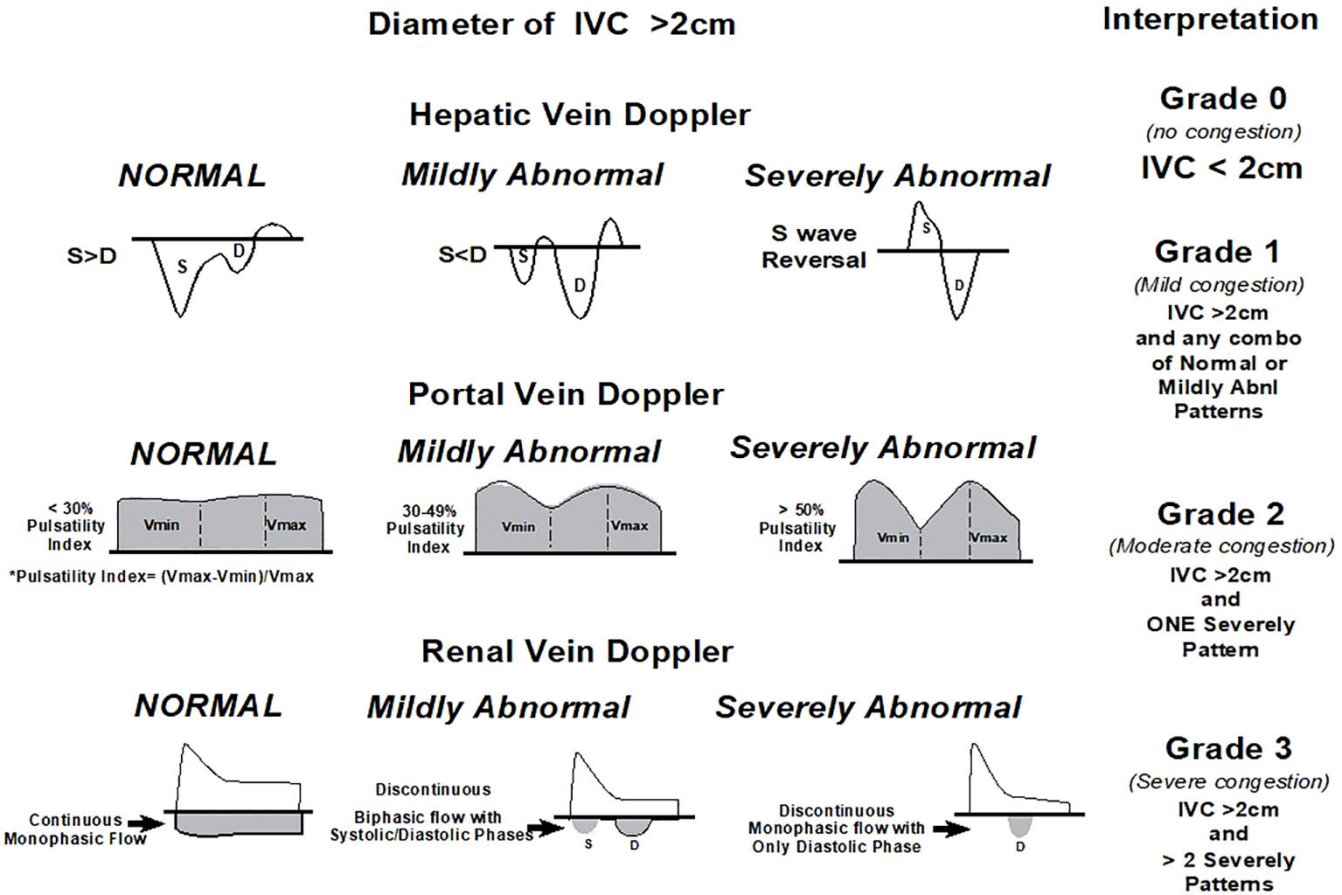
Venous excess Doppler ultrasound grading. Based on the degree of anomalies on the hepatic, portal, and intrarenal venous Doppler, three degrees of congestion are identified when the diameter of the IVC is greater than 2 cm. When the S wave on a hepatic vein Doppler is smaller than the D wave but remains below the baseline, it is classified as mildly abnormal; when the S wave is inverted, it is classified as seriously abnormal. When the pulsatility is between 30% and 50%, the portal vein Doppler is deemed slightly abnormal; when it is ≥50%, it is deemed seriously abnormal. * Pulsatility measurement points. When an intrarenal vein Doppler has a distinct D and S component and is pulsatile, it is considered mildly aberrant; when it shows a monophasic D-only pattern, it is considered highly abnormal. With permission, this figure was modified from NephroPOCUS.com. VExUS, venous excess Doppler ultrasonography; IVC, inferior vena cava.

Traditional methods of assessing fluid status and cardiac function often fall short when attempting to accurately identify venous congestion, a condition that can lead to organ dysfunction and worsened prognosis in critically ill patients (59). The venous excess ultrasound (VExUS) score emerges as a promising tool in this context. VExUS is an ultrasound-based scoring system that assesses venous congestion by examining the IVC, hepatic vein (HV), portal vein (PV), and intrarenal venous (IRV) Doppler waveforms (**Figure 5**). This approach provides a more comprehensive understanding of the patient’s resuscitation strategy, incorporating the concept of fluid tolerance (FT) (60). VExUS can guide clinicians in making more informed decisions regarding fluid administration, deresuscitation, and potentially preventing the progression of kidney dysfunction. Recent studies incorporating VExUS into critical care protocols have shown that patients with reduced scores over 48 h and higher doses of diuretics had significantly more RRT-free days over a 28-day period (61). The ability of VExUS to non-invasively and dynamically assess venous congestion offers a significant advantage over traditional hemodynamic monitoring techniques, which often fail to detect subtle changes in venous return and congestion. The application of VExUS has been associated with the prognostication of AKI in patients with cardiorenal syndrome, aiding in the clinical decision for the patient to undergo fluid removal (62). Furthermore, VExUS-guided fluid management has the potential to personalize and optimize fluid therapy, moving beyond the “one-size-fits-all” approach (63). This personalized management is particularly beneficial in the heterogeneous population of critically ill patients, where the physiological responses to illness and therapy can vary markedly.

The renal resistive index (RRI), assessed via Doppler ultrasound, plays a pivotal role in nephrology, particularly in critically ill patients for the early detection and management of AKI and hepatorenal syndrome (HRS). RRI reflects renal vascular resistance by measuring the ratio of (peak systolic velocity–end diastolic velocity) to peak systolic velocity within the renal arteries. Elevated RRI values signify increased vascular resistance and reduced renal perfusion, which are early indicators of renal dysfunction in conditions such as sepsis, shock, or other critical illnesses.

In the context of AKI, RRI monitoring allows for preemptive intervention before changes in traditional biomarkers like serum creatinine are evident. This capability is crucial in guiding therapeutic strategies to optimize renal perfusion and mitigate further renal damage. For instance, in septic shock, elevated RRI values prompt adjustments in fluid resuscitation and vasopressor therapy to maintain adequate renal blood flow, potentially preventing AKI progression (64).

In hepatorenal syndrome, where renal impairment occurs secondary to severe liver disease and circulatory dysfunction, RRI serves as a vital tool in assessing renal vascular resistance changes. In HRS, elevated RRI values often indicate decreased renal perfusion due to systemic vasodilation and renal vasoconstriction. Monitoring RRI guides therapeutic decisions such as albumin infusions, vasoconstrictor therapy (e.g., terlipressin), or consideration of liver transplantation to improve renal function (65).

The continuous assessment of RRI offers dynamic insights into renal hemodynamics, crucial for managing critically ill patients prone to renal complications. Its integration into clinical practice enhances diagnostic precision, facilitates early intervention, and optimizes patient outcomes by tailoring therapeutic approaches to individual renal perfusion dynamics.

## 6 Abdominal ultrasonography for nephrologists

In AKI, the use of abdominal ultrasound helps in differentiating pre-renal, renal, and post-renal causes. It can identify obstructions in the urinary tract, such as stones or clots, and can also detect conditions like hydronephrosis. It can also guide percutaneous procedures, like kidney biopsies or the placement of dialysis catheters, reducing the risk of complications. Furthermore, abdominal ultrasonography is beneficial in screening for renal malignancies and in the follow-up of renal transplant patients. It can monitor graft size, detect complications like collections or obstruction, and assess vascular anastomoses.

The contribution of venous congestion to kidney dysfunction is increasingly being acknowledged, as unresolved congestion is linked to unfavorable kidney outcomes in patients with heart failure (66). Similarly, any condition that results in elevated central venous pressure, such as pulmonary hypertension, can lead to impaired kidney perfusion by increasing cardiac afterload. POCUS allows for clinicians to objectively evaluate hemodynamics at the bedside, thereby guiding patient management. Although inferior vena cava (IVC) POCUS is employed to estimate right atrial pressure, it cannot demonstrate organ congestion and bears several limitations, including the influence of ventilation settings, the patient’s inspiratory efforts, coexisting cardiac conditions, and intra-abdominal hypertension (67). Recently, venous excess Doppler ultrasound has emerged as a real-time tool to assess venous congestion at the organ level (68). Severe flow abnormalities in hepatic, portal, and kidney parenchymal veins have been shown to predict the risk of congestive kidney injury and aid in monitoring the efficacy of decongestive therapy (69). Herein, we provide a brief overview of the various components of venous excess Doppler ultrasound and share our perspective on incorporating this innovative tool in nephrology practice.

## 7 Ultrasound assessment of arteriovenous fistulas in nephrology practice

Assessing the patency and functionality of arteriovenous (AV) fistulas is critical for nephrologists managing patients with end-stage renal disease reliant on hemodialysis. Ultrasound has become indispensable in this regard, offering a non-invasive and real-time method to evaluate AV fistulas. Using Doppler ultrasound, nephrologists can assess important hemodynamic parameters such as flow velocity and resistance indices and detect abnormalities such as stenosis or thrombosis (70).

Regular ultrasound surveillance plays a pivotal role in the early detection of complications like aneurysm formation or pseudoaneurysms, which could compromise the patency of AV fistulas. This proactive approach allows for timely intervention to maintain vascular access integrity, ensuring effective hemodialysis delivery (71).

In clinical practice, nephrologists rely on ultrasound-guided assessments to optimize dialysis efficacy and minimize access-related complications, thereby improving overall patient outcomes and quality of life.

## 8 Limitations of multi-organ POCUS

Patient-specific variables, including adiposity, subcutaneous edema, and the presence of inserted devices, which are prevalent in critically ill patients, can substantially impede the precision and practicality of POCUS. These circumstances can diminish the quality of ultrasound images, making it difficult to acquire dependable evaluations. Moreover, the assessment of POCUS results necessitates a considerable degree of expertise and proficiency, which may not be evenly accessible in all clinical environments, resulting in inconsistencies in the precision of diagnoses and treatment choices. Furthermore, within the framework of RRT, the dynamic alterations in fluid distribution and fluctuations in cardiovascular function present additional obstacles to the reliable analysis and verification of ultrasound images over a period of time. We must combine the data and acknowledge that they are not a substitute for the comprehensive information obtained from a meticulous clinical examination and other diagnostic modalities. Though POCUS is a useful and quickly deployable tool that does not require invasive procedures, its limits highlight the importance of thorough training, establishing standardized protocols, and incorporating other clinical data to enhance patient care.

## 9 Conclusions

Hemodynamic instability is a frequent condition in critically ill patients undergoing RRT and is associated with increased mortality rates and the potential impairment of renal recovery. While excessive ultrafiltration is a known cause of hypotension, it may not always constitute the primary underlying mechanism. Multiple other RRT-related factors can contribute to a decreased cardiac output, decreased peripheral resistance, or both. In the management of patients requiring RRT, POCUS plays a crucial role in the initial diagnostic assessment of hemodynamically unstable patients, as well as in monitoring the outcomes of therapeutic interventions. We strongly advocate the incorporation of POCUS into the standard of care for all nephrologists and urge hospitals to provide the necessary resources to support a successful program.
